# Barriers to initiating SGLT2 inhibitors in diabetic kidney disease: a real-world study

**DOI:** 10.1186/s12882-021-02381-3

**Published:** 2021-05-14

**Authors:** Su Jin Jeong, Seung Eun Lee, Dong Hyun Shin, Ie Byung Park, Hui Seung Lee, Kyoung-Ah Kim

**Affiliations:** 1 Department of Internal Medicine, Sejong Hospital, Bucheon, Korea; 2 Department of Internal Medicine, Dongguk University Ilsan Hospital, Goyang, Korea; 3 Department of Internal Medicine, Bundang Jesaeng Hospital, Seongnam, Korea; 4 Division of Endocrinology and Metabolism, Department of Internal Medicine, Gachon University Gil Medical Center, Incheon, Korea; 5 Department of Biostatistics, School of Medicine, Dongguk University, Goyang, Korea

**Keywords:** Diabetes Mellitus, Type 2, Diabetic Nephropathies, Renal Insufficiency, Chronic, Sodium-Glucose Transporter 2

## Abstract

**Background:**

Sodium-glucose cotransporter 2 inhibitor (SGLT2i) should be considered for patients with type 2 diabetes (T2D) and chronic kidney disease (CKD) having estimated glomerular filtration rate (eGFR) ≥ 30 mL/min/1.73 m^2^ and urine albumin-to-creatinine ratio (UACR) > 30 mg/g. However, SGLT2i is currently underprescribed among eligible, at-risk patients for CKD progression. We analyzed prescription patterns and barriers to initiating SGLT2i in patients with T2D and CKD in real practice.

**Methods:**

A total of 3,703 consecutive outpatients with T2D from four teaching hospitals during six months (2019 ~ 2020) were reviewed. Five eGFR categories (G1, ≥ 90; G2, 60–89; G3ab, 30–59; G4-5, < 30 mL/min/1.73 m^2^) and three UACR categories (A1, < 30; A2, 30–300; A3, > 300 mg/g) were used to define CKD status.

**Results:**

Overall, 25.8 % patients received SGLT2i in the following eGFR and albuminuria categories: G1 (A1, 31 %; A2, 48 %; A3, 45 %); G2 (A1, 18 %; A2, 24 %; A3, 30%); and G3 (A1, 9 %; A2, 7 %; A3, 13 %).

Total prevalence estimate of CKD was 33.8 % (*n* = 1,253), of whom 25.6 % patients received SGLT2i. We defined eGFR ≥ 45 mL/min/1.73 m^2^ and UACR ≥ 30 mg/g as high-risk CKD group eligible for SGLT2i (*n* = 905), of whom 32.9 % patients were treated with an SGLT2i. In this high-risk group, SGLT2i initiation showed negative correlations with age ≥ 65 years and recent hospitalization. Conversely, HbA1c level, body mass index (BMI), presence of diabetic retinopathy, and previous heart failure events were positively correlated with SGLT2i initiation.

**Conclusions:**

Only 32.9 % of T2D with CKD eligible for SGLT2i is currently treated with SGLT2i in real-world clinical practice. The older patient group and clinical inertia are the main barriers to initiate SGLT2i for eligible patients. Clinicians should change the glucocentric approach and focus on reducing renal events in T2D.

**Supplementary Information:**

The online version contains supplementary material available at 10.1186/s12882-021-02381-3.

## Background

Chronic kidney disease (CKD) attributed to diabetes (diabetic kidney disease) occurs in 20–40 % of patients with diabetes [1, 2]. Diabetic kidney disease (DKD) is a clinical diagnosis made based on the presence of albuminuria and/or reduced estimated glomerular filtration rate (eGFR) [2]. It is caused by hyperglycemia, hypertension, aging, and other risk factors of chronic kidney disease. Although the prevalence of DKD is increasing, there are few medications to treat or slow its disease course.

Sodium-glucose cotransporter 2 inhibitor (SGLT2i) is a renoprotective glucose-lowering drug [2, 3]. Renin-angiotensin-aldosterone system inhibitor (RAASi) is effective in protecting against the progression of nephropathy due to type 2 diabetes (T2D) [4, 5]. American Diabetes Association (ADA) recommends that the use of an SGLT2i for patients with T2D and DKD having an eGFR ≥ 30 mL/min/1.73 m^2^ and urinary albumin-to-creatinine ratio (UACR) > 30 mg/g, particularly in those with UACR > 300 mg/g [2].

However, real-world evidence demonstrates that SGLT2i is currently underutilized for eligible patients and that the decision to start SGLT2i is typically deferred to the endocrinologist [6–8]. Although recent studies have reported that the use of SGLT2i reached 6.2 % in T2D and CKD, they did not clarify the barriers against initiation of SGLT2i, including clinical factors such as the impact of glycemic control [9, 10].

Thus, the objective of this study was to determine the proportion of patients with T2D who received SGLT2i and factors limiting the initiation of SGLT2i in patients with eGFR ≥ 45 mL/min/1.73 m^2^ and UACR ≥ 30 mg/g in diabetes clinics from teaching hospitals.

## Methods

### Study subjects

The present study was based on data obtained from four teaching hospitals in Seoul Metropolitan Area from September 2019 to May 2020 because drug labeling for SGLT2i was extended to eGFR ≥ 45 mL/min/1.73m^2^ in August 2019 after the CREDENCE trial released in June 2019 [3]. We excluded patients with systemic conditions that might affect vascular glomerulonephritis or vasculitis, patients with other reasons of renal dysfunction, and patients with acute kidney injury (AKI).

Of 4,186 consecutive subjects visiting each diabetes clinic, subjects who were prescribed SGLT2i from outside hospitals (*n* = 74), those who used glucagon-like peptide − 1 receptor agonist (GLP-1 RA) before initiation of SGLT2i (*n* = 163), and adults without measurements of serum creatinine levels (*n* = 57) or UACR (*n* = 189) were excluded. After these exclusions, 3,703 subjects were included in the final analysis (Fig. S1). This retrospective cross-sectional study was approved by the Institutional Review Board (IRB) of Dongguk University Ilsan Hospital and participating hospitals (IRB No. 2020-03-052), and informed consent was waived.

### Demographic, anthropometric, and clinical characteristics

To analyze prescription patterns according to CKD status, patients were divided into two groups: SGLT2i nonusers and SGLT2i initiators. Clinical characteristics were collected based on the time of the last outpatient department visit. However, for SGLT2i initiators, age, body mass index (BMI), diabetes duration, duration of atherosclerotic cardiovascular disease or heart failure (collectively called CVD-HF), and recent hospitalization data were collected based on the time of SGLT2i initiation. The definition of recent hospitalization was hospital admission within one year of the investigation, excluding minor procedure hospitalization (for the SGLT2i initiators, before initiation of SGLT2i).

Comorbidities were determined based on chart review. We defined previously documented history of atherosclerotic cardiovascular diseases (ASCVDs), including coronary artery diseases (CAD; chronic coronary syndrome, acute coronary syndrome and/or coronary artery revascularization), cerebrovascular diseases (cerebral infarction and/or transient ischemic attack), peripheral artery diseases (peripheral artery occlusive disease and/or lower limb revascularization), and heart failure (HF) according to the International Classification of Diseases (ICD–10). The occurrence of diabetic retinopathy (DR) was determined by an ophthalmologist. Medication histories regarding anti-hypertensive drugs including RAASi and lipid-lowering drugs were also reviewed. For glucose-lowering drugs, data of nonusers were based on the last prescription. Glucose-lowering drugs of SGLT2i initiators were based on the last prescription just before switching to SGLT2i.

### Laboratory data

Most current laboratory data of subjects were recorded within two years if available. For SGLT2i initiators, HbA1c and eGFR values were based on the time of SGLT2i initiation. Lab data were obtained from each hospital.

eGFR was calculated from the serum creatinine level standardized to IDMS using the Chronic Kidney Disease-Epidemiology Collaboration (CKD-EPI) equation [11].

We used five eGFR categories (G1, ≥ 90; G2, 60–89; G3a, 45–59; G3b, 30–44; G4-5, < 30 mL/min/1.73 m^2^) [12, 13]. There were three albuminuria categories: A1, < 30 mg/g; A2, 30–300 mg/g; and A3 > 300 mg/g. Here, A2 was defined when at least two abnormal UACR 30–300 mg/g had been present regardless of interval, continuity, or number of total measurements. A3 was defined as one elevated UACR > 300 mg/g. Therefore, CKD was defined as an eGFR < 60 mL/min/1.73 m^2^ or an UACR ≥ 30 mg/g. In this study, high-risk CKD group eligible for SGLT2i was defined as an eGFR ≥ 45 mL/min/1.73 m^2^ and UACR **≥** 30 mg/g [2].

### Statistical analysis

Patients initiating SGLT2i (SGLT2i initiators) during the study period were compared with SGLT2i nonusers. These two groups were mutually exclusive. Data were described as mean ± SD or as percentages and number of cases. Student’s t-test or Kruskal-Wallis one-way analysis of variance was used for continuous variables while Pearson Chi-square test was used for categorical variables. Predictive factors for SGLT2i initiation were obtained using univariate and multivariate logistic regression analyses. Analyzed results were described as odds ratios with their 95 % confidence intervals. Statistical significance was considered when p value was less than 0.05. All statistical analyses were performed using R Statistical Software version 4.0.2 (R Foundation for Statistical Computing, Vienna, Austria).

## Results

### Baseline characteristics of the total study population

**Fig. 1:**
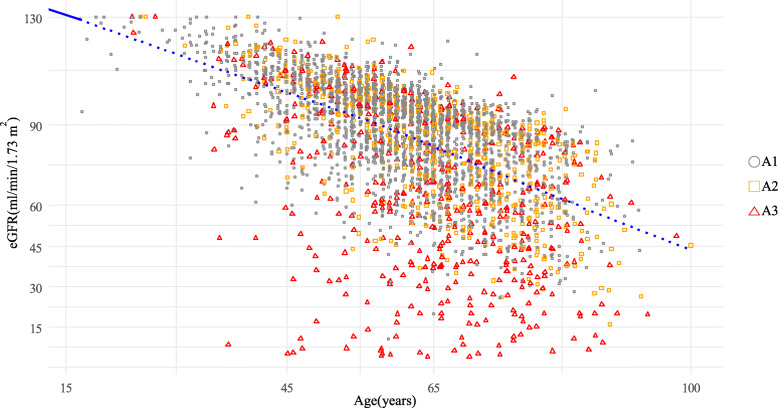
Relationship between age and estimated glomerular filtration rate (eGFR) by albuminuria. Pearson coefficient between age and eGFR was r = -0.57 (*p* < 0.001). Blue dotted line represents correlation line. A1 ~ A3 denote albuminuria categories: A1, < 30 mg/g; A2, 30 ~ 300 mg/g; A3, > 300 mg/g

**Fig. 2:**
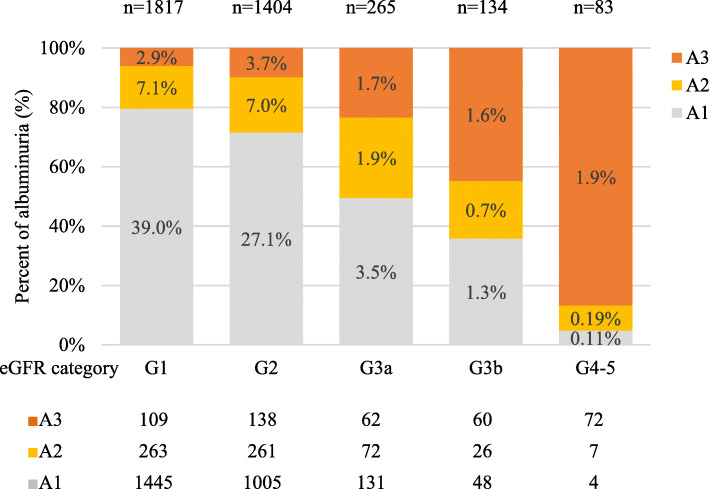
Albuminuria categories in relation to eGFR category. The stacked bar graph shows percentages of patients in A1, A2, and A3 albuminuria categories with respect to each eGFR category. Numbers over columns indicate the number of patients within each eGFR category. Percentage figures in the columns represent relative proportions of cases in the entire study population. The table below the graph shows absolute number of patients in each category. The group indicated by the dotted line in the figure is the high-risk CKD group eligible for SGLT2i in this study. A1-A3 denote albuminuria category. Albuminuria categories: A1, < 30 mg/g; A2, 30 ~ 300 mg/g; A3, > 300 mg/g. G1-G5 denote GFR categories: G1, ≥ 90 mL/min/1.73 m^2^; G2, 60-89mL/min/1.73m^2^;G3a,45-59mL/min/1.73m^2^;G3b,30–44mL/min/1.73m^2^;G4-5,<30 mL/min/1.73 m^2^

Baseline demographic and clinical characteristics of the total study population are presented in Table S1. We enrolled 3,703 study subjects (2,205 men and 1,498 women), of whom 25.8 % were treated with an SGLT2i. Their mean age was 61.4 ± 12.0 years. Approximately 39.1 % of patients were older than 65 years (*n* = 1,448). The mean BMI was 26.0 ± 3.8 kg/m^2^, with 57.7 % of patients classified as obese (BMI ≥ 25 kg/m^2^) [14]. The prevalence of diagnosed CVD-HF was 33 %. The mean value of eGFR was 84.8 ± 21.7 mL/min/1.73 m^2^. eGFR was negatively correlated with aging (Fig. 1). Proportions of categories classified based on eGFR and albuminuria are shown in Fig. 2. Prevalent rates of eGFR categories G1, G2, G3a, G3b, and G4-5 were 49.1 %, 37.9 %, 7.2 %, 3.6 %, and 2.2 %, respectively. A2 albuminuria was present in 17.0 % and A3 was present in 11.9 % of subjects. Total prevalence estimate of CKD was 33.8 % (*n* = 1,253). According to eGFR and albuminuria categories, the prevalence of CKD was as follows: G1 and A2-3, 10.0 %; G2 and A2-3, 10.7 %; G3a and A1-3, 7.2 %; G3b and A1-3, 3.6 %; and G4-5 and A1-3, 2.2 % (Fig. 2). The prevalence of CKD was higher in older subjects than in younger subjects: 46.9 % in the elderly group (age ≥ 65 years), 25.5 % in the middle-aged group (age, 45–64 years), and 25.4 % in the young group (age < 45 years). The prevalence of CKD was higher in subpopulations with diagnosed CVD-HF (43.6 % of 1223 subjects).

### SGLT2i prescription pattern of the total study population

To characterize SGLT2i initiators (25.8 %, *n* = 956), the population was divided into SGLT2i nonusers vs. SGLT2i initiators. In the elderly group, 16 % were SGLT2i initiators (*n* = 240). Overall, 25.6 % of T2D with CKD are currently treated with SGLT2i. In G4-G5 categories, only one patient received SGLT2i.

**Fig. 3:**
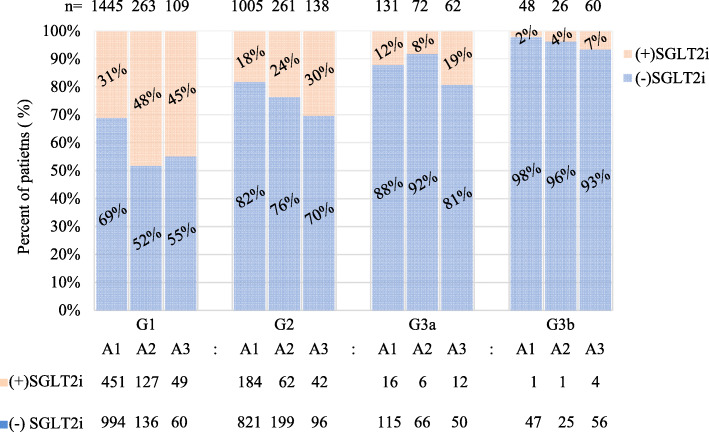
Proportions of SGLT2i initiators each eGFR and albuminuria categories. Numbers over columns indicate the number of patients within each eGFR and albuminuria category. Percentage figures in columns represent proportions of patients with SGLT2i initiators (+) or nonusers (-) from top to bottom. The table below the graph shows the absolute number of patients in each category. A1-A3 denote albuminuria categories: A1, < 30 mg/g; A2, 30 ~ 300 mg/g; A3, > 300 mg/g. G1-G3a denote GFR categories. GFR categories: G1, ≥ 90 mL/min/1.73 m^2^; G2, 60–89 mL/min/1.73 m^2^; G3a,45–59 mL/min/1.73 m^2^

In each eGFR category with albuminuria status (A1; A2; A3), proportions of SGLT2 initiators were as follows: G1 (31 %; 48 %; 45 %), G2 (18 %; 24 %; 30 %), G3a (12 % ;8 %; 19 %), and G3b (2 % ;4 %; 7 %) (Fig. 3). For initiators, most people were in G1-G2 categories (95.7 %). Practically, SGLT2i was allowed in eGFR category ≤ G3a (eGFR ≥ 45 mL/min/1.73 m^2^) in Korea. In eGFR categories G1-G3a (*n* = 3,486), SGLT2i initiators accounted for 27.2 % (*n* = 949).

### SGLT2i initiation in the high-risk CKD group eligible for SGLT2i

We defined eGFR ≥ 45 mL/min/1.73 m^2^ and UACR **≥** 30 mg/g as a high-risk CKD group eligible for SGLT2i (G1-2A2; G3aA2, or G1-2A3; G3aA3**)**. The high-risk cohort was composed of 905 adults with diabetes (24.4 % of the entire study population), of whom 32.9 % (*n* = 298) were treated with an SGLT2i. However, in the case of the elderly, the high-risk group consisted of 28.6 % subjects, of which 17.6 % received treatment with SGLT2i (*n* = 73).

By eGFR category, the prevalence of high-risk CKD was as follows: G1, 41.1 % (*n* = 372); G2, 44.1 % (*n* = 399); and G3a, 14.8 % (*n* = 134) (Fig. 2 with dotted box group).

The SGLT2i initiator group had higher eGFR (90.7 ± 18.2 vs. 79.7 ± 18.9 mL/min/1.73 m^2^; *p* < 0.001) and higher concentration of HbA1c (8.0 ± 1.2 vs.7.3 ± 1.3 % ; *p* < 0.001) than the nonuser group (Table 1). SGLT2i initiators were younger (56.5 ± 10.5 vs. 65.8 ± 12.3; *p* < 0.001) and more obese (BMI: 28.3 ± 3.9 vs. 25.4 ± 3.5 kg/m^2^; *p* < 0.001). SGLT2i initiators had shorter diabetes duration (10.2 ± 7.5 vs. 13.3 ± 8.8 years; *p* < 0.001) and CVD-HF duration (5.8 ± 4.8 vs. 7.4 ± 5.7 years; *p* = 0.007).

**Table 1:** Baseline demographic and clinical characteristics of the high-risk CKD group eligible for SGLT2i

SGLT2i	Overall(*n* = 905)	Nonusers(*n* = 607)	Initiators(*n* = 298)	*p*-value
Age, yr	63.2 ± 12.2	65.8 ± 12.3	56.5 ± 10.5	< 0.001
Male gender	558 (61.7 %)	365 (60.1 %)	193 (64.8 %)	0.202
BMI, kg/m^2^	26.4 ± 3.9	25.4 ± 3.5	28.3 ± 3.9	< 0.001
Duration of diabetes, yr	12.3 ± 8.5	13.3 ± 8.8	10.2 ± 7.5	< 0.001
HbA1c, %	7.6 ± 1.3	7.3 ± 1.3	8.0 ± 1.2	< 0.001
eGFR, mL/min/1.73 m^2^	83.4 ± 19.3	79.7 ± 18.9	90.7 ± 18.2	< 0.001
eGFR category				< 0.001
G1	372 (41.1 %)	196 (32.3 %)	176 (59.1 %)	
G2	399 (44.1 %)	295 (48.6 %)	104 (34.9 %)	
G3a	134 (14.8 %)	116 (19.1 %)	18 (6.0 %)	
Albuminuria category				0.911
A2	596 (65.9 %)	401 (66.1 %)	195 (65.4 %)	
A3	309 (34.1 %)	206 (33.9 %)	103 (34.6 %)	
Diabetic retinopathy				0.019
No	492 (54.4 %)	345 (56.8 %)	147 (49.3 %)	
Yes	284 (31.4 %)	172 (28.3 %)	112 (37.6 %)	
Not available	129 (14.3 %)	90 (14.8 %)	39 (13.1 %)	
SBP, mm Hg	131.2 ± 15.0	131.0 ± 15.6	131.8 ± 13.9	0.435
DBP, mm Hg	75.6 ± 9.8	75.0 ± 9.8	76.9 ± 9.6	0.007
LDL-C, mg/dL	71.5 ± 24.3	72.5 ± 24.5	69.6 ± 24.0	0.094
Triglyceride, mg/dL	150.2 ± 124.5	145.7 ± 98.3	159.6 ± 165.2	0.181
HDL-C, mg/dL	48.2 ± 17.2	48.1 ± 16.3	48.4 ± 18.8	0.818
Cancer	65 (7.2 %)	55 (9.1 %)	10 (3.4 %)	0.003
Recent hospitalization	150 (16.6 %)	117 (19.3 %)	33 (11.1 %)	0.003
CVD-HF	339 (37.5 %)	221 (36.4 %)	118 (39.6 %)	0.391
Heart failure	26 (2.9 %)	7 (1.2 %)	19 (6.4 %)	< 0.001
Stroke	113 (12.5 %)	92 (15.2 %)	21 (7.0 %)	0.001
CAD	219 (24.2 %)	128 (21.1 %)	91 (30.5 %)	0.002
PAOD	23 (2.5 %)	19 (3.1 %)	4 (1.3 %)	0.167
Duration of CVD-HF, yr	6.8 ± 5.5	7.4 ± 5.7	5.8 ± 4.8	0.007
SGLT2i initiation year				< 0.001
2015		0 (0.0 %)	45 (15.1 %)	
2016		0 (0.0 %)	52 (17.4 %)	
2017		0 (0.0 %)	47 (15.8 %)	
2018		0 (0.0 %)	82 (27.5 %)	
2019		0 (0.0 %)	57 (19.1 %)	
2020		0 (0.0 %)	15 (5.0 %)	
not applicable		607 (100.0 %)	0 (0.0 %)	
Medication (%)				
Metformin	837 (92.5 %)	554 (91.3 %)	283 (95.0 %)	0.05
Insulin	220 (24.3 %)	128 (21.1 %)	92 (30.9 %)	0.008
RAASi	641 (70.8 %)	416 (68.5 %)	225 (75.5 %)	0.066
Statins	813 (89.8 %)	532 (87.6 %)	281 (94.3 %)	< 0.001

Values are presented as mean ± standard deviation or number (%)

*ASCVD* atherosclerotic cardiovascular disease, *BMI* body mass index, *CVD-HF* atherosclerotic cardiovascular disease or heart failure, *DBP* diastolic blood pressure, *eGFR* estimated glomerular filtration rate, *HbA1c* glycosylated hemoglobin, *HDL-C *high-density lipoprotein cholesterol, *HF* heart failure, *LDL-C* low-density lipoprotein cholesterol, *RAASi* renin-angiotensin-aldosterone system inhibitor, *SBP *systolic blood pressure, *SGLT2i* sodium-glucose cotransporter-2 inhibitor

SGLT2i initiators had comparable rates of previous composite CVD-HF events (39.6 % vs. 36.4 %; *p* = 0.391). However, SGLT2i initiators had higher rates of HF (6.4 % vs. 1.2 %; *p* < 0.001) and CAD (30.5 % vs. 21.1 %; *p* = 0.002) than nonusers. In contrast, the SGLT2i initiator group had lower stroke events than the control group (7.0 % vs. 15.2 %; *p* = 0.001).

Cancer patients or recently hospitalized patients were less likely to be started on an SGLT2i, but patients with diabetic retinopathy were more likely to be started on an SGLT2i(37.6 vs. 28.3 %; *p* = 0.019). Likewise, the SGLT2i initiator group had comparable treatment rates with RAASi (75.5 vs. 68.5 %; *p* = 0.066) but higher insulin use rates before switching to SGLT2i (30.9 vs. 21.1 %; *p* = 0.008).

### Factors related to failure to start SGLT2i in those with high-risk CKD group eligible for SGLT2i

Logistic regression analyses revealed that SGLT2i initiation had negative correlations with age ≥ 65 years and recent hospitalization in the high-risk group. Conversely, SGLT2i initiation was positively correlated with HbA1c level, BMI, presence of DR, and previous HF events (Table 2).

**Table 2:** Results of logistic regression analysis to determine variables associated with SGLT2i initiation according to baseline characteristics of study participants in the high-risk CKD group eligible for SGLT2i

		CRUDE			ADJUST		
Variable	OR	95 % CI	*p*-value	OR	95 % CI	*p*-value	
Age							
17–44(reference)							
45–64	1.2027	0.95–1.52	0.1260	1.3581	1.02–1.81	0.0384	
>=65	0.3282	0.25–0.43	**< 0.0001**	0.4068	0.28–0.60	**< 0.0001**	
Female sex	0.9229	0.79–1.08	0.3190	1.0209	0.83–1.26	0.8491	
BMI, kg/m^2^	1.2654	1.21–1.33	**< 0.0001**	1.2723	1.20–1.35	**< 0.0001**	
DM duration (per year)	0.9564	0.94–0.98	**< 0.0001**	0.9806	0.92–1.04	0.5203	
DM duration, yr							
< 5 (reference)							
5 ~ 9	1.3008	0.99–1.71	0.0601	1.1535	0.78–1.71	0.4759	
10 ~ 14	0.8289	0.64–1.08	0.1637	0.9427	0.67–1.33	0.7388	
> 15	0.6581	0.51–0.84	**0.0009**	0.9195	0.47–1.82	0.8090	
HbA1c	1.5200	1.34–1.72	**< 0.0001**	1.4504	1.24–1.70	**< 0.0001**	
Albuminuria categories							
A2(reference)							
A3	0.9796	0.84–1.15	0.8000	0.9213	0.75–1.14	0.4460	
Diabetic retinopathy	1.2183	1.04–1.42	**0.0122**	1.4757	1.18–1.84	**0.0005**	
Cancer	0.5636	0.38–0.83	**0.0033**	0.6862	0.43–1.11	0.1224	
Recent hospitalization	0.6951	0.55–0.87	**0.0017**	0.6007	0.44–0.82	**0.0011**	
CVD-HF	1.0782	0.92–1.26	0.3380	0.7312	0.41–1.32	0.2980	
Heart failure	2.6806	1.62–4.44	**< 0.0001**	5.1875	2.56–10.49	**< 0.0001**	
Stroke	0.6422	0.49–0.85	**< 0.0001**	0.9431	0.57–1.57	0.8221	
CAD	1.2914	1.09–1.53	**< 0.0001**	1.9390	1.10–3.40	**0.0211**	
PAOD	0.7000	0.40–1.22	0.2063	1.0210	0.48–2.18	0.9572	

Bold values denote statistical significance at the *p* < 0.05 level

*ASCVD* atherosclerotic cardiovascular disease, *BMI* body mass index, *CAD* coronary artery disease, *CI* confidence interval, *CVD-HF* atherosclerotic cardiovascular disease or heart failure, *DBP* diastolic blood pressure, *HbA1c* glycosylated hemoglobin, *HDL-C *high-density lipoprotein cholesterol, *HF* heart failure, *LDL-C* low-density lipoprotein cholesterol, *OR* odds ratio, *PAOD *peripheral arterial occlusive disease, *RAASi* renin*-*angiotensin-aldosterone system inhibitors, *SBP* systolic blood pressure, *SGLT2i *sodium-glucose cotransporter-2 inhibitor

Elderly patients were less likely to start an SGLT2i (OR:0.41, 95 % CI: 0.28–0.60 vs. 17–44 years). Recently hospitalized patients were less likely to start an SGTLT2i (OR: 0.60, 95 % CI: 0.44–0.82). On the contrary, high HbA1c levels increased the odds of SGLT2i initiation (OR: 1.45, 95 % CI: 1.24–1.70). High BMI increased the odds of SGLT2i initiation (OR: 1.27, 95 % CI: 1.20–1.35). The presence of DR increased the odds of SGLT2i initiation (OR: 1.48, 95 % CI: 1.18–1.84). HF patients were also more likely to start an SGLT2i (OR: 5.19, 95 % CI: 2.56–10.49).

### Trends in baseline characteristics of patients initiating an SGLT2 inhibitor in the high-risk CKD group eligible for SGLT2i

Characteristics of patients receiving SGLT2i changed over time (Table S2). Annual SGLT2i prescription was increased gradually, peaking in 2018 (27.5 %). The most notable change was the proportion of patients newly initiated on an SGLT2i in CVD-HF, increasing from 22.2 % to 2015 to 43.9 % in 2019 (*p* = 0.001). The proportion of patients with CAD was increased from 15.6 % to 2015 to 33.3 % in 2019 (*p* = 0.004). The proportion of patients with stroke was similarly increased from 4.4 % to 2015 to 12.3 % in 2019 (*p* = 0.012).

Another substantial change was an increase in patients’ mean age of initiating SGLT2i therapy, increasing from 52.3 ± 9.7 years in 2015 to 60.1 ± 10.7 years in 2019 (*p* = 0.004). The duration of diabetes increased from 9.2 ± 5.6 years in 2015 to 13.0 ± 9.2 years in 2019 (*p* = 0.008). The proportion of patients with A3 was similarly increased from 35.6 % to 2015 to 73.3 % in 2020 (*p* = 0.014). When we combined G3a and G3b, the proportion of patients newly initiated on an SGLT2i in G3 category was increased from 6.7 % to 2015 to 16.2 % in 2019 (*p* = 0.026, data not shown).

## Discussion

In this study of real-world data with T2D, SGLT2i was initiated most often for patients with the lowest risk (i.e., young, G1-G2 categories). SGLT2i was initiated for only 32.9 % of patients in the high-risk CKD group eligible for SGLT2i for renal protection (G1-G3a with A2-A3 categories). The proportion of patients who received SGLT2i in the high-risk group was significantly lower in G3a (14.8 %) than in G1 (41.4 %) and G2 (44.1 %) category. Such lower usage of SGLT2i was contrary to the 2020 ADA/EASD (European Association for the Study of Diabetes) consensus guideline to add SGLT2i for patients with CKD (eGFR 30 to ≤ 60 mL/min/1.73 m^2^ or UACR > 30 mg/g, particularly UACR > 300 mg/g) [15].

In general, among T2D individuals, 50–65 %, 20–30 %, and 15–25 % have no DKD, albuminuric DKD with preserved eGFR, and reduced eGFR, respectively [16]. In a USA study, the prevalence of CKD was 43.5 % in the T2D population overall and 61.0 % in those aged ≥ 65 years [17]. In this study, total prevalence of CKD was estimated to be 33.8 % overall and 46.9 % in those aged ≥ 65 years. Our study had more patients in G3-G5 categories (13.1 %) and A3 category (11.8 %) than those in a Korean nationwide survey. In the KNHANES 2011–2013 on diabetes, the total prevalence of CKD was estimated to be 27.6 %. For each eGFR category, prevalence results were: G1, 45.2 %; G2, 45.1 %; G3, 8.8 %; and G4-5, 0.9 % [18]. Albuminuria prevalence was: A1,77.7 %; A2,18.3 %; and A3,4.0 %.

In our study, 8.5 % of patients in G3-G5 categories initiated SGLT2i. On the contrary, in an Australian study regarding T2D and G3-G5 CKD, SGLT2i use was 6.2 % [9]. In a USA study, 7.2 % of patients with diabetes overall initiated an SGLT2i regardless of their CKD stage. In that report, diabetic nephropathy was present in 12.7 % of SGLT2i users and 16.1 % of nonusers. CKD was present in 6.7 % of SGLT2i users and 11.7 % of nonusers [10]. However, the definition of chronic kidney disease or diabetic nephropathy was not clear. These low percentages of SGLT2i use in T2D and CKD were similar to T2D and cardiovascular disease (CVD) data. For high-risk CVD patients eligible for recent cardiovascular outcome trials, only 5.2 % received SGLT2i in the USA and 11.1 % in the UK [19, 20]. Since the permitted prescribing range for SGLT2i is for those with eGFR ≥ 45 mL/min/1.73 m^2^ in Korea, patients with G3b category have no opportunity to receive SGLT2i. However, there is an additional limitation of starting SGLT2i for those in eGFR ≥ 45 mL/min/1.73 m^2^ and UACR **≥** 30 mg/g.

This study indicated that patients with SGLT2i initiators were younger than SGLT2i nonusers in the high-risk CKD group eligible for SGLT2i. In the elderly group, the prevalence of high-risk CKD eligible for SGLT2i was higher (28.6 %) than that in those aged < 65 years (21.8 %). However, older patients were less likely to start SGLT2i therapy (17.6 %) than those aged < 65 years (45.8 %) in the high-risk group. Trials in the SGLT2i class show that older patients have similar or greater benefits than younger patients [21–23]. These mutually opposed results are based on ADA recommendations that glycemic goals for some older adults might reasonably be relaxed as part of individualized care [24]. Since older patients are more likely to have various complications, they are cautious about using newly available drugs. Besides, while understanding of clinical benefits of SGLT2i is evolving, side effects such as volume depletion might be more common for older patients [24, 25]. It is also difficult to distinguish whether chronic kidney disease in the elderly is due to aging or diabetes, although the DAPA-CKD study has shown that dapagliflozin can reduce the risk of worsening kidney function or death in patients with CKD without T2D [26]. Notably, for subjects older than 65 years without albuminuria, CKD has been proposed as eGFR below 45 mL/min/1.73 m² to distinguish age-related from disease-related changes in eGFR [27].

There were HbA1c differences in SGLT2i initiation. This is due to clinical inertia of physicians not to change medications when glycemic control is in the target range despite the presence of CVD-HF or CKD [6]. This is contrary to the ADA/EASD consensus recommending SGLT2i should be considered independently of baseline HbA1c or individualized HbA1c target in appropriate high-risk individuals with established T2D [15]. Furthermore, those who received SGLT2i showed significantly higher eGFR, BMI, and shorter diabetes duration. Indeed, it has been reported that obesity is a greater driver of treatment options when comparing SGLT2i, GLP-1 RA, and dipeptidyl peptidase 4 inhibitors [28]. Efforts to provide treatment following guidelines and extension of an SGLT2i ‘s official prescription drug label for eGFR may help improve renal outcomes of patients with T2D.

Trends in clinical characteristics for SGLT2i have also demonstrated potential impacts of clinical practice guidelines or formulary information on rates of SGLT2i initiation [29, 30]. In fact, the presence of HF and CAD significantly increased the odds of SGLT2i initiation. Drug labeling for eGFR to start SGLT2i also influenced the rise in prescription. In Korea, SGLT2i was introduced in 2014 and approved for those with eGFR ≥ 60 ml/min/1.73m^2^. Empagliflozin 10 mg and dapagliflozin were approved in 2015 and 2019, respectively, for those with eGFR ≥ 45 ml/min/1.73m^2^. Hence, we can notice the rise of SGLT2i prescriptions in G3 category recently. Notably, safety and efficacy results from the DAPA-CKD trial can broaden the population of patients eligible for SGLT2i to those in the upper range of stage 4 CKD [26]. Physicians should identify patients with T2D who may benefit from renal function protection by using SGLT2i.

The strength of the present study was that it addressed underutilized SGLT2i data in the real-world for less renal benefit control among outpatients with CKD in diabetes. Our study was conducted at four diabetes centers with a larger sample size in a considerably more diverse and complex outpatient population (including G4-G5 category) than previous studies. Furthermore, it uses CKD data from Korea where the prevalence of CKD and CVD in patients with T2D is expected to be different from that in a randomized clinical trial or the West. Besides, we examined the impact of glycemic control and clinical characteristics on the likelihood of SGLT2i initiation.

Our study has some limitations. First, the study sample was not representative of the Korean population. Therefore, results should be interpreted with caution. Its main limitations derive from its retrospective cross-sectional observational nature. Since GLP-1 RA is considered a renoprotective glucose-lowering drug, we excluded GLP-1 RA users before initiation of SGLT2i in this study (3.9 %). The determination of the presence of chronic kidney disease based on eGFR was made using a single random sample of laboratory values [31]. The albuminuria category was based on two elevated UACR levels regardless of the continuity or interval. This condition might have overestimated the proportion of A2 category since increased albuminuria should be confirmed on repeat testing over 3 to 6 months. Besides, we started the study shortly after the publication of CREDENCE trial that showed renal benefits of SGLT2i in patients with eGFR > 30 mL/min/1.73 m^2^ [3]. Therefore, if the study was started late, more patients with CKD would have initiated SGLT2i.

## Conclusions

In the high-risk CKD group eligible for SGLT2i, younger patients with poor glycemic control in eGFR G1-G2 categories received more SGLT2i than in those without each corresponding characteristic. Thus, physicians should be aware of limitations of this glucocentric approach and focus on a personalized approach to CKD in diabetes management. Also, health plans supporting these evidence-based treatment strategies could improve renal outcomes of patients with this disease.

To overcome therapeutic inertia, the medical community needs several improvements. First, an accurate diagnosis of chronic kidney disease is necessary in order to identify eligible patients. Second, regulatory authorities should extend the eGFR values above 30 mL/min/1.73 m^2^. Finally, since more than half of CKD patients are older adults in this study, conservative clinical guidelines on the treatment of T2D for the elderly should be individualized according to CKD status.

## Supplementary information

**Additional file 1: Table S1.** Baseline demographic and clinical characteristics of the total study population. **Table S2.** Trends of SGLT2i initiation in the high-risk CKD group eligible for SGLT2i. **Figure S1.** Flow diagram of study subjects.

## Data Availability

The datasets used and/or analyzed during the current study are available from the corresponding author on reasonable request.

## Abbreviations

AKI: Acute kidney injury
ADA: American Diabetes Association
ASCVD: Atherosclerotic cardiovascular disease
BMI: Body mass index
CAD: Coronary artery diseases
CKD: Chronic kidney disease
CKD-EPI: Chronic Kidney Disease-Epidemiology Collaboration
CVD: Cardiovascular disease
CVD-HF: Cardiovascular disease or heart failure
DKD: Diabetic kidney disease
DR: Diabetic retinopathy
EASD: European Association for the Study of Diabetes
eGFR: Estimated glomerular filtration rate
GLP-1 RA: Glucagon-like peptide − 1 receptor agonist
HF: Heart failure
RAASi: Renin-angiotensin-aldosterone system inhibitor
SGLT2i: Sodium-glucose cotransporter 2 inhibitor
T2D: Type 2 diabetes
UACR: Urine albumin-to-creatinine ratio
