# Supplementary material for: Barriers to initiating SGLT2 inhibitors in diabetic kidney disease: a real-world study
Source: BMC Nephrol. 2021 May 14;22:177. doi: 10.1186/s12882-021-02381-3 (PMC8122538; doi:10.1186/s12882-021-02381-3)

**Table S1** Baseline demographic and clinical characteristics of the total study population

| SGLT2i | Overall (n = 3703) | Nonusers (n = 2747) | Initiators (n = 956) | *p*-value |
| --- | --- | --- | --- | --- |
| Age, yr | 61.4 ± 12.0 | 63.4 ± 11.8 | 55.6 ± 10.5 | < 0.001 |
| Male gender | 2205 (59.5%) | 1643 (59.8%) | 562 (58.8%) | 0.605 |
| BMI, kg/m^2^ | 26.0 ± 3.8 | 25.3 ± 3.4 | 28.2 ± 4.1 | < 0.001 |
| Duration of diabetes, yr | 10.2 ± 8.0 | 10.8 ± 8.2 | 8.5 ± 7.0 | < 0.001 |
| HbA1c, % | 7.3 ± 1.2 | 7.2 ± 1.2 | 7.9 ± 1.2 | < 0.001 |
| eGFR, mL/min/1.73 m^2^ | 84.8 ± 21.7 | 81.9 ± 22.3 | 93.2 ± 17.1 | < 0.001 |
| eGFR category |  |  |  | <0.001 |
| G1 | 1817 (49.1%) | 1190 (43.3%) | 627 (65.6%) |  |
| G2 | 1404 (37.9%) | 1116 (40.6%) | 288 (30.1%) |  |
| G3a | 265 (7.2%) | 231 (8.4%) | 34 (3.6%) |  |
| G3b | 134 (3.6%) | 128 (4.7%) | 6 (0.6%) |  |
| G4 | 50 (1.4%) | 49 (1.8%) | 1 (0.1%) |  |
| G5 | 33 (0.9%) | 33 (1.2%) | 0 (0.0%) |  |
| Albuminuria category |  |  |  | 0.003 |
| A1 | 2633 (71.1%) | 1981 (72.1%) | 652 (68.2%) |  |
| A2 | 629 (17.0%) | 433 (15.8%) | 196 (20.5%) |  |
| A3 | 441 (11.9%) | 333 (12.1%) | 108 (11.3%) |  |
| Diabetic retinopathy |  |  |  | 0.001 |
| No | 2422 (65.4%) | 1786 (65.0%) | 636 (66.5%) |  |
| Yes | 730 (19.7%) | 520 (18.9%) | 210 (22.0%) |  |
| Not available | 551 (14.9%) | 441 (16.1%) | 110 (11.5%) |  |
| SBP, mm Hg | 129.0 ± 14.2 | 129.1 ± 14.2 | 128.6 ± 13.9 | 0.32 |
| DBP, mm Hg | 75.0 ± 9.7 | 74.7 ± 9.7 | 75.9 ± 9.7 | 0.001 |
| LDL-C, mg/dL | 72.1 ± 24.5 | 72.7 ± 24.7 | 70.3 ± 23.8 | 0.01 |
| Triglyceride, mg/dL | 140.7 ± 108.4 | 136.9 ± 98.1 | 151.7 ± 133.1 | 0.002 |
| HDL-C, mg/dL | 49.1 ± 14.4 | 49.3 ± 14.4 | 48.8 ± 14.5 | 0.364 |
| Cancer | 287 (7.8%) | 241 (8.8%) | 46 (4.8%) | < 0.001 |
| Recent hospitalization | 569 (15.4%) | 461 (16.8%) | 108 (11.3%) | < 0.001 |
| CVD-HF | 1223 (33.0%) | 838 (30.5%) | 385 (40.3%) | < 0.001 |
| Heart failure | 105 (2.8%) | 60 (2.2%) | 45 (4.7%) | < 0.001 |
| Stroke | 374 (10.1%) | 316 (11.5%) | 58 (6.1%) | < 0.001 |
| CAD | 842 (22.7%) | 531 (19.3%) | 311 (32.5%) | < 0.001 |
| PAOD | 55 (1.5%) | 44 (1.6%) | 11 (1.2%) | 0.402 |
| Duration of CVD-HF, yr | 6.5 ± 5.5 | 7.0 ± 5.7 | 5.4 ± 4.8 | < 0.001 |
| SGLT2i initiation year |  |  |  | < 0.001 |
| 2014 | 4 (0.1%) | 0 (0.0%) | 4 (0.4%) |  |
| 2015 | 167 (4.5%) | 0 (0.0%) | 167 (17.5%) |  |
| 2016 | 171 (4.6%) | 0 (0.0%) | 171 (17.9%) |  |
| 2017 | 136 (3.7%) | 0 (0.0%) | 136 (14.2%) |  |
| 2018 | 220 (5.9%) | 0 (0.0%) | 220 (23.0%) |  |
| 2019 | 201 (5.4%) | 0 (0.0%) | 201 (21.0%) |  |
| 2020 | 57 (1.5%) | 0 (0.0%) | 57 (6.0%) |  |
| not applicable | 2747 (74.2%) | 2747 (100.0%) | 0 (0.0%) |  |
| Medication (%) |  |  |  |  |
| Metformin | 3233 (87.3%) | 2358 (85.8%) | 875 (91.5%) | < 0.001 |
| Insulin | 638 (17.2%) | 430 (15.7%) | 208 (21.8%) | < 0.001 |
| RAASi | 2027 (54.7%) | 1418 (51.6%) | 609 (63.7%) | < 0.001 |
| Statins | 3242 (87.6%) | 2356 (85.8%) | 886 (92.7%) | < 0.001 |

Values are presented as mean ± standard deviation or number (%).

ASCVD, atherosclerotic cardiovascular disease; BMI: body mass index; CAD, coronary artery disease; CVD-HF, atherosclerotic cardiovascular disease or heart failure ; DBP, diastolic blood pressure; eGFR, estimated glomerular filtration rate; HbA1c, glycosylated hemoglobin; HDL-C, high-density lipoprotein cholesterol; HF, heart failure; LDL-C, low-density lipoprotein cholesterol; PAOD, peripheral arterial occlusive disease; RAASi, renin-angiotensin-aldosterone system inhibitor; SBP, systolic blood pressure; SGLT2i, sodium-glucose cotransporter-2 inhibitor.

**Table S2.** Trends of SGLT2i initiation in the high-risk CKD group eligible for SGLT2i

| SGLT2i initiation year | 2015  (n = 45) | 2016  (n = 52) | 2017  (n = 47) | 2018  (n = 82) | 2019  (n = 57) | 2020  (n = 15) | *p*-value |
| --- | --- | --- | --- | --- | --- | --- | --- |
| Age, yr | 52.3 ± 9.7 | 55.6 ± 9.9 | 55.2 ± 10.4 | 56.7 ± 10.7 | 60.1 ± 10.7 | 60.4 ± 8.5 | **0.004** |
| Male gender | 26 (57.8%) | 36 (69.2%) | 30 (63.8%) | 56 (68.3%) | 39 (68.4%) | 6 (40.0%) | 0.283 |
| BMI, kg/m2 | 29.8 ± 3.5 | 28.9 ± 4.6 | 28.3 ± 3.7 | 27.8 ± 3.6 | 27.6 ± 3.9 | 27.7 ± 2.8 | **0.032** |
| Duration of diabetes, yr | 9.2 ± 5.6 | 9.6 ± 6.5 | 8.8 ± 6.0 | 9.4 ± 7.3 | 13.0 ± 9.2 | 13.8 ± 9.9 | **0.008** |
| HbA1c, % | 7.9 ± 1.1 | 8.2 ± 1.3 | 8.0 ± 1.1 | 8.0 ± 1.2 | 7.9 ± 1.4 | 8.3 ± 1.2 | 0.64 |
| eGFR, | 95.2 ± 20.0 | 93.9 ± 19.3 | 92.0 ± 15.6 | 90.1 ± 17.8 | 86.1 ± 17.3 | 83.9 ± 17.7 | 0.063 |
| eGFR category |  |  |  |  |  |  | 0.664 |
| G1 | 30 (66.7%) | 34 (65.4%) | 30 (63.8%) | 45 (54.9%) | 29 (50.9%) | 8 (53.3%) |  |
| G2 | 12 (26.7%) | 16 (30.8%) | 16 (34.0%) | 32 (39.0%) | 23 (40.4%) | 5 (33.3%) |  |
| G3a | 3 (6.7%) | 2 (3.8%) | 1 (2.1%) | 5 (6.1%) | 5 (8.8%) | 2 (13.3%) |  |
| Albuminuria category | |  |  |  |  |  | **0.014** |
| A2 | 29 (64.4%) | 38 (73.1%) | 36 (76.6%) | 51 (62.2%) | 37 (64.9%) | 4 (26.7%) |  |
| A3 | 16 (35.6%) | 14 (26.9%) | 11 (23.4%) | 31 (37.8%) | 20 (35.1%) | 11 (73.3%) |  |
| Diabetic retinopathy | |  |  |  |  |  | **0.023** |
| No | 25 (55.6%) | 31 (59.6%) | 27 (57.4%) | 39 (47.6%) | 20 (35.1%) | 5 (33.3%) |  |
| Yes | 17 (37.8%) | 18 (34.6%) | 16 (34.0%) | 33 (40.2%) | 21 (36.8%) | 7 (46.7%) |  |
| Not available | 3 (6.7%) | 3 (5.8%) | 4 (8.5%) | 10 (12.2%) | 16 (28.1%) | 3 (20.0%) |  |
| Cancer | 1 (2.2%) | 1 (1.9%) | 1 (2.1%) | 4 (4.9%) | 1 (1.8%) | 2 (13.3%) | 0.272 |
| Recent hospitalization | 3 (6.7%) | 2 (3.8%) | 6 (12.8%) | 7 (8.5%) | 13 (22.8%) | 2 (13.3%) | **0.03** |
| CVD-HF | 10 (22.2%) | 13 (25.0%) | 29 (61.7%) | 36 (43.9%) | 25 (43.9%) | 5 (33.3%) | **0.001** |
| Heart failure | 1 (2.2%) | 0 (0.0%) | 2 (4.3%) | 10 (12.2%) | 5 (8.8%) | 1 (6.7%) | 0.065 |
| Stroke | 2 (4.4%) | 0 (0.0%) | 0 (0.0%) | 10 (12.2%) | 7 (12.3%) | 2 (13.3%) | **0.012** |
| CAD | 7 (15.6%) | 13 (25.0%) | 25 (53.2%) | 23 (28.0%) | 19 (33.3%) | 4 (26.7%) | **0.004** |
| PAOD | 1 (2.2%) | 0 (0.0%) | 2 (4.3%) | 0 (0.0%) | 1 (1.8%) | 0 (0.0%) | 0.372 |
| Duration of CVD-HF, yr | 8.3 ± 5.2 | 7.0 ± 5.3 | 6.1 ± 4.7 | 4.8 ± 3.9 | 5.3 ± 5.6 | 5.4 ± 4.6 | 0.363 |

Values are presented as mean ± standard deviation or number (%). Bold values denote statistical significance at the p < 0.05 level.

ASCVD, atherosclerotic cardiovascular disease; BMI: body mass index; CAD, coronary artery disease; CVD-HF, atherosclerotic cardiovascular disease or heart failure ; DBP, diastolic blood pressure; eGFR, estimated glomerular filtration rate(mL/min/1.73 m2); HbA1c, glycosylated hemoglobin; HDL-C, high-density lipoprotein cholesterol; HF, heart failure; LDL-C, low-density lipoprotein cholesterol; PAOD, peripheral arterial occlusive disease; RAASi, renin-angiotensin-aldosterone system inhibitor; SBP, systolic blood pressure; SGLT2i, sodium-glucose cotransporter-2 inhibitor.

**Fig.S1** Flow diagram of study subjects. eGFR, estimated glomerular filtration rate (eGFR); GLP-1 RA, Glucagon-like peptide **-1 receptor agonist;** N/A, not available; SGLT2i, sodium-glucose cotransporter-2 inhibitor; T2D, type 2 diabetes.


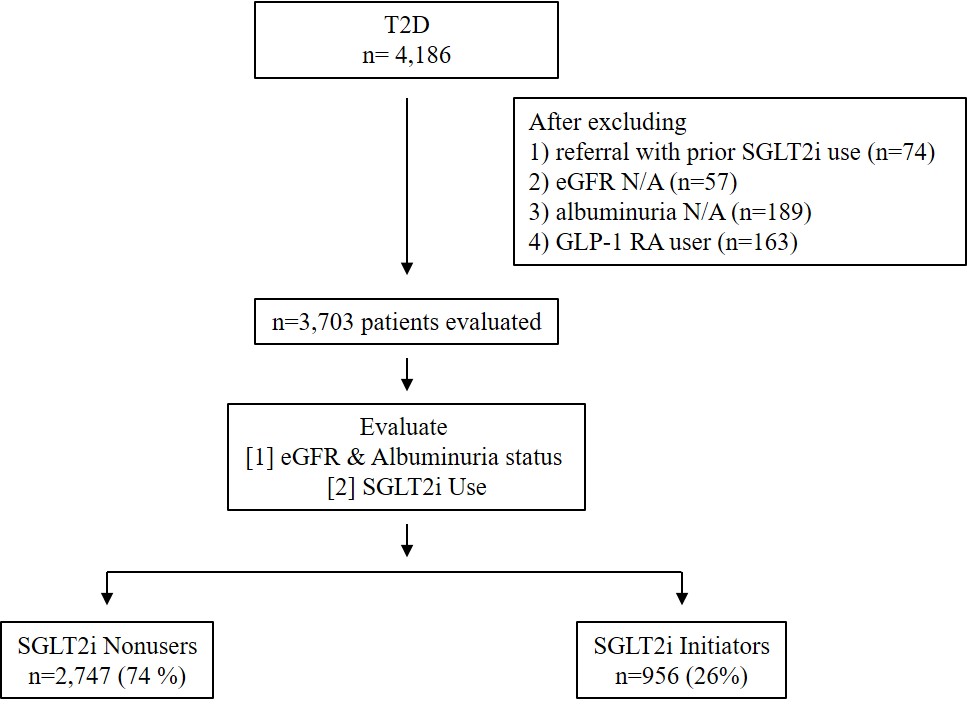

Supplement: Supplementary file 1 — Additional file 1: Table S1. Baseline demographic and clinical characteristics of the total study population. Table S2. Trends of SGLT2i initiation in the high-risk CKD group eligible for SGLT2i. Figure S1. Flow diagram of study subjects. [file 12882_2021_2381_MOESM1_ESM.docx]
